# Effect of Different Doses of Atipamezole on Reversal of Medetomidine-Induced Tear-Flow Decrease in Rats

**DOI:** 10.3390/vetsci7040197

**Published:** 2020-12-03

**Authors:** Teppei Kanda, Manami Gotoh, Ayumi Makino, Kayo Furumoto, Yuki Shimizu, Takamasa Itoi, Noritaka Maeta, Toshinori Furukawa

**Affiliations:** 1 Faculty of Veterinary Medicine, Okayama University of Science, 1-3 Ikoino-oka, Imabari, Ehime 794-8555, Japan; k-furumoto@vet.ous.ac.jp (K.F.); y-shimizu@vet.ous.ac.jp (Y.S.); t-itoi@vet.ous.ac.jp (T.I.); n-maeta@vet.ous.ac.jp (N.M.); 2 Department of Comparative Animal Science, College of Life Science, Kurashiki University of Science and the Arts, 2640 Nishinoura, Tsurajima-cho, Kurashiki, Okayama 712-8505, Japan; vet.anesth.analg+mgotoh@gmail.com (M.G.); vet.anesth.analg+amakino@gmail.com (A.M.); tfurukaw@mac.com (T.F.); 3 Department of Animal Pharmaceutical Science, School of Pharmaceutical Science, Kyushu University of Health and Welfare, 1714-1 Yoshino-cho, Nobeoka, Miyazaki 882-8508, Japan

**Keywords:** α_2_-adrenoceptor agonist, medetomidine, atipamezole, tear flow, rat

## Abstract

It has been reported that α_2_-adrenoceptor agonists such as medetomidine decrease tear flow in many species, including rats. Few studies have investigated the involvement of α_2_-adrenoceptor in decreased tear flow; the issue has not been illustrated sufficiently. Therefore, we aimed to investigate the effect of different doses of atipamezole on the reversal of medetomidine-induced tear-flow decrease to reveal the specific involvement of α_2_-adrenoceptor. Treatment with 400, 800, or 1600 µg/kg atipamezole (or saline as the control) was intramuscularly administered to rats 15 min following intramuscular administration of 200 µg/kg medetomidine. After medetomidine administration, tear flow was measured using a phenol red thread test (PRTT). PRTT values decreased significantly after 200 µg/kg medetomidine administration. The PRTT values after 800 (optimal dose to reverse) and 1600 µg/kg atipamezole administration reached baseline, but never exceeded it significantly. Treatment with 400 µg/kg atipamezole also reversed the decrease in PRTT value but the PRTT remained lower than baseline. The optimal dose and the higher dose of atipamezole fully reversed the medetomidine-induced decrease in tear flow to the baseline level in rats, while the lower dose of atipamezole partially recovered tear flow.

## 1. Introduction

Medetomidine, a representative α_2_-adrenoceptor agonist, is widely used for sedation or analgesia of many species in veterinary medicine [1,2,3,4]. Not only do α_2_-adrenoceptor agonists, including medetomidine, act as sedatives or analgesics to relieve animals from fear, anxiety, or pain, they also attenuate excessive neurohormonal reaction to the noxious stimulus, which contributes to maintaining homeostasis [5,6,7,8]. On the other hand, α_2_-adrenoceptor agonists such as medetomidine, dexmedetomidine, detomidine, xylazine, and clonidine have been reported to decrease tear flow in many animal species, including dogs, cats, horses, pigs, and rats [9,10,11,12,13,14,15,16,17,18]. Insufficient tear flow may cause structural or functional issues on the ocular surface, including the cornea, which induces pain or irritation [19,20,21]. Thus, a decrease in tear flow induced by α_2_-adrenoceptor agonists may develop into a serious issue that spoils animal welfare.

In several studies, the involvement of α_2_-adrenoceptor in tear-flow decrease has been reported; however, the detailed mechanism is not yet clear [11,12,14,15,17,18,22,23]. A limited number of reports have suggested that α_2_-adrenoceptor plays a role in the decrease of tear flow. However, one study could not deny the possibility that the α_2_-adrenoceptor antagonist itself increased tear flow, regardless of the antagonizing mechanism involved, because only a limited range of doses of the antagonist was used [9]. Another study could not exclude the involvement of α_1_-adrenoceptor because the investigation involved a non-specific α-adrenoceptor antagonist [16]. To reveal the involvement of α_2_-adrenoceptor in tear-flow decrease, it is necessary to indicate that more specific α_2_-adrenoceptor antagonists, such as atipamezole, reverse the tear-flow decrease caused by the α_2_-adrenoceptor agonist [24].

Therefore, we aimed to investigate the effects of different doses of atipamezole on the reversal of medetomidine-induced tear-flow decrease to reveal the specific involvement of α_2_-adrenoceptor. In the present study, we hypothesized that the optimal dose of atipamezole would fully reverse the tear-flow decrease caused by the α_2_-adrenoceptor agonist.

## 2. Materials and Methods

### 2.1. Animals

Male Slc: Wistar/ST rats (Rattus norvegicus) (*n* = 32, aged 8–11 weeks; Japan SLC, Inc., Hamamatsu, Japan) weighing 321 ± 24 g (mean ± standard deviation) were used. The animals were allowed to acclimatize for a week before treatment and were housed in groups of two to three animals in individually ventilated cages (NIKI SHOUJI Co., Tokyo, Japan) placed in one room. A 12:12 h light-dark cycle (light period, 8:00 a.m. to 8:00 p.m.) was maintained, the room temperature was between 22 and 26 °C, and humidity was 30–60%. Water and a commercially pelleted diet (CE-2; CLEA Japan, Inc., Tokyo, Japan) were provided ad libitum. After the intramuscular administration of medetomidine, a single rat was placed in an individually ventilated cage on the laboratory table of the experimental room; the rat stayed there for 2 h between measurements. Wood shavings (CL-4161; CLEA Japan, Inc., Tokyo, Japan) were used as bedding material in all cages. Eight rats were assigned randomly to one of four atipamezole treatment groups and were not blinded. All procedures were approved by the Animal Care and Use Committee of Kurashiki University of Science and the Arts (approval number: 26-02).

### 2.2. Drug Treatment

Medetomidine (1000 µg/mL, Domitor; Nippon Zenyaku Kogyo Co., Ltd., Fukushima, Japan) at a dose of 200 µg/kg was administered intramuscularly to rats in all groups. Fifteen minutes after medetomidine administration, atipamezole (5000 µg/mL, Antisedan; Nippon Zenyaku Kogyo Co., Ltd., Fukushima, Japan) at doses of 400, 800, or 1600 µg/kg or 0.32 mL/kg saline solution (equal volume to 1600 µg/kg atipamezole) was administered. According to the treatment received, groups were named MA400, MA800, MA1600, and control groups, respectively. The drug solution was injected into the caudal part of the left thigh using a microsyringe (1/2 mL BD Lo-Dose Insulin Syringe 29 G × 1/2 inch; BD, Franklin Lakes, NJ, USA) at 10:00 a.m.

### 2.3. Measurement

Tear flow was measured using a phenol red thread tear test (Zone-Quick; AYUMI Pharmaceutical Corporation, Tokyo, Japan) in four rats for the left eye and four rats for the right eye, with a total of eight rats in each group [25,26]. The 3 mm tips of threads were placed on the medial canthus for 15 s. The length of the moistened area from the edge was measured as the phenol red thread test (PRTT) value. Tear flow was measured approximately 1 min before (Pre) and 5, 10, 15, 20, 25, 30, 45, and 60 min after medetomidine administration.

### 2.4. Statistical Analysis

PRTT data were reported as the mean ± standard error of the mean (SEM). PRTT values were confirmed for normality of distribution using the Shapiro–Wilk test. The effect of time on each treatment and the treatment effect at each time point were evaluated using two-way analysis of variance for repeated measures. When a significant difference was found, the Tukey test was used to compare the mean PRTT value at each time point with the baseline value and the mean value at 15 min after medetomidine administration within each group. The differences in mean PRTT values between groups at each time point were also analyzed using the Tukey test. The area under the curve (AUC) of PRTT values (0 mm/15s) from 25 to 60 min after medetomidine administration was calculated. AUC data are reported as the mean ± standard deviation (SD). The AUC between groups was compared using the Kruskal–Wallis test. When a significant difference was found, Dunn’s test was used to compare the mean AUCs.

All statistical analyses were performed using GraphPad Prism 8 (GraphPad Software, San Diego, CA, USA). A *p*-value < 0.01 was considered statistically significant.

## 3. Results

**Figure 1:**
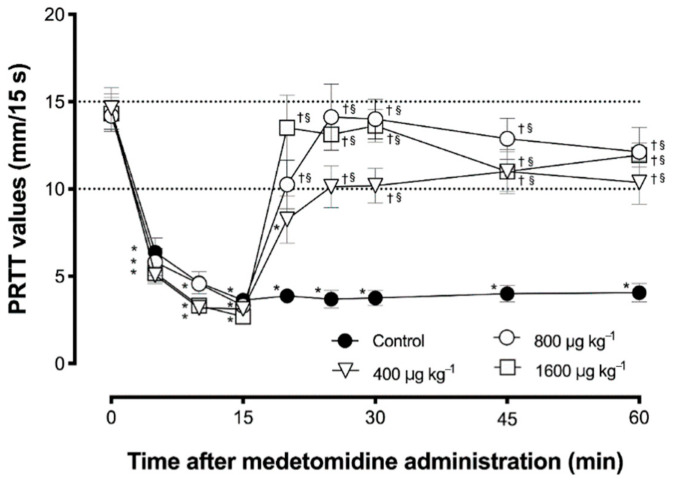
The mean ± standard error of the mean of phenol red thread test (PRTT) values before and after intramuscular administration of medetomidine (*n* = 8). Statistical differences with significance (*p* < 0.01) compared with (*) the baseline value within the group, (†) the value at each time point in the control group, and (§) the value at 15 min after medetomidine administration within the group.

In all groups, PRTT values decreased significantly at 5, 10, and 15 min after administration of 200 µg/kg medetomidine (Figure 1). In the control group, a significant decrease lasted until the end of the observation period. From 5 to 55 min after administration, 800 and 1600 µg/kg atipamezole significantly reversed the decrease in PRTT compared to the value 15 min after medetomidine administration in each group. In both groups, there were no significant differences between the reversed PRTT values and baseline values. PRTT values were significantly higher for 40 min compared with those of the control group at each time point in both the MA800 and MA1600 groups. The PRTT values after 800 or 1600 µg/kg atipamezole administration reached the baseline level but did not exceed it significantly during observation. In the MA800 and MA1600 groups, RRTT values, in which the decrease was reversed by atipamezole, tended to decrease again at 30 and 45 min after atipamezole administration, without statistical significance. While 400 µg/kg atipamezole also showed a tendency to reverse the decrease in PRTT value at 5 min after administration; however, the PRTT value was significantly lower than the baseline value at that time. In the MA400 group, no significant difference was found between the PRTT value at 5 min after atipamezole administration and the baseline value, despite the reversal tendency of the mean value.

From 10 to 45 min after atipamezole administration, there were no significant differences between the PRTT value and the baseline in the MA400 group, and the PRTT value was significantly higher than that in the control group. In the MA400 group, unlike the MA800 and MA1600 groups, the PRTT value did not reach the baseline level after atipamezole administration; however, it did not show a tendency toward a decrease in PRTT value.

**Figure 2:**
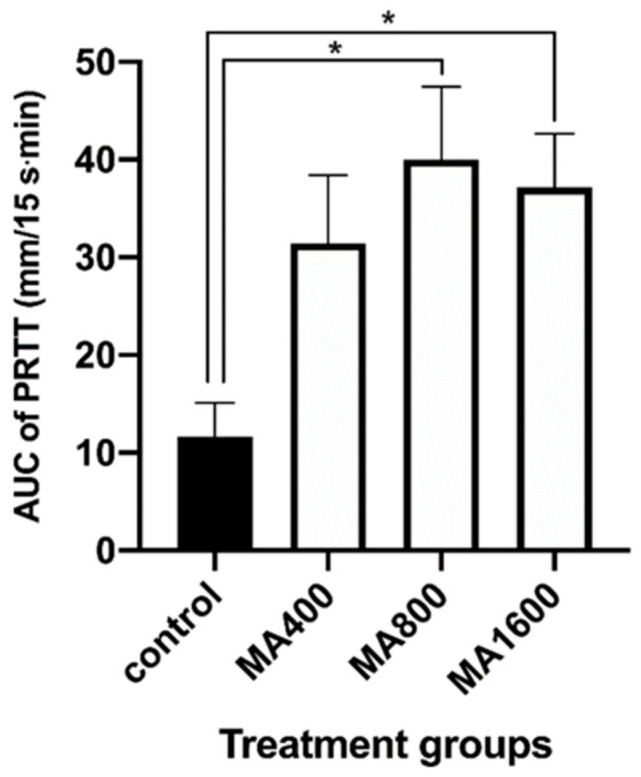
The mean ± standard deviation of the area under the curve (AUC) of phenol red thread test (PRTT) values from 25 to 60 min after medetomidine administration (*n* = 8). (*) Statistical differences with significance (*p* < 0.01) compared with the control group.

The AUC of the MA800 and MA1600 groups from 10 to 45 min after atipamezole administration, when the PRTT values were significantly reversed in all treated groups compared with the values at 15 min after medetomidine administration, were significantly larger than that of the control group (Figure 2). However, no statistically significant difference was found between the AUCs of the MA400 and control groups (*p* = 0.11).

## 4. Discussion

Medetomidine significantly decreased rat tear flow measured by PRTT in the present study. These results agree with those of our previous report on rats and of studies on other species [9,11,15,17,18]. In dogs, it was reported that administration of atipamezole at a dose five times higher than that of medetomidine, administered 15 min before, reversed the decrease in tear flow caused by medetomidine in dogs [9]. The optimal dose of atipamezole in dogs was described as a dose four-to-six times higher than that of medetomidine to reverse effects such as sedation [1,27,28]. In rats, a dose approximately three-to-five times higher than that of atipamezole was used to reverse the effect of medetomidine [29,30,31]. Therefore, in the present study, a dose of atipamezole four times higher than that of medetomidine was regarded as the optimal dose. In this study, 800 µg/kg atipamezole, which was determined to be the optimal dose, reversed the decrease in tear flow caused by 200 µg/kg medetomidine and recovered tear flow to baseline level within 10 min after administration. In both rats and dogs, atipamezole reversed the tear-flow decrease caused by medetomidine.

Sanchez et al. reported the possibility that the decrease and subsequent increase in tear flow after medetomidine and atipamezole administration may be caused by the effect of medetomidine, which was reversed by atipamezole, such as vasoconstriction of the tear gland [9]. However, Sanchez et al. could not exclude the possibility that atipamezole itself increased tear flow, regardless of the administration of medetomidine, because they investigated only the effect of the optimal-to-lower dose of atipamezole, which was about 2.5-to-5 times higher than that of medetomidine [9]. Weisse et al. stated that pretreatment with phentolamine, a non-specific α-adrenoceptor antagonist, blocked the decrease in tear flow caused by clonidine, an α_2_-adrenoceptor agonist similar to medetomidine [16]. Weisse et al. could not rule out the involvement of α_1_-adrenoceptor, because phentolamine is a non-specific antagonist for both α_1_- and α_2_-adrenoceptor. In the present study, the tear-flow decrease was reversed to baseline level faster in the group administered 1600 µg/kg atipamezole, twice the optimal dose, than in the optimal-dose group, though it was not reduced significantly beyond the baseline level. Thus, even though atipamezole was administered at an excessive dose, it only reversed the tear flow decrease to baseline. This result suggested that atipamezole itself did not increase tear flow dose-dependently, regardless of the medetomidine-induced decrease in tear flow, although further studies are needed to reveal the detailed effects of various doses of atipamezole administered alone.

At 400 µg/kg atipamezole, half of the optimal dose, the tear flow decrease was reversed but more slowly than in the group treated with 800 µg/kg atipamezole. In the group administered 400 µg/kg, the tear flow never reached the baseline level during the observation period, although the tear flow reached the baseline level 5 min after 1600 µg/kg atipamezole administration and 10 min after 800 µg/kg atipamezole administration. Meanwhile, there were no significant differences in PRTT values at each time point among the groups administered 400, 800, and 1600 µg/kg atipamezole. The AUCs 15–60 min after medetomidine administration in the groups treated with 800 and 1600 µg/kg atipamezole were significantly larger than that of the control group, although no significant difference in AUC was found between the control group and the group administered 400 µg/kg atipamezole. These results indicate that atipamezole at a dose less than the optimal dose could not reverse the decrease in tear flow caused by medetomidine administration.

In the present study, we revealed that atipamezole, a specific α_2_-adrenoceptor antagonist, reversed the tear-flow decrease caused by medetomidine administration. In addition, it was also showed that atipamezole itself did not increase tear flow in a dose-dependent manner within the tested doses. These findings indicated that the medetomidine-induced decrease in tear flow was caused by mechanisms specifically involved with the α_2_-adrenoceptor.

In the groups administered 800 and 1600 µg/kg atipamezole, the PRTT values tended to relapse into a decrease after the reversed PRTT value reached the baseline level, without statistical significance. Previously, Vähä-Vahe and Vainio reported that some dogs relapsed into sedation after medetomidine-induced sedation was reversed with atipamezole [27,28]. In a study by Nishimura et al. on pigs sedated with medetomidine combined with midazolam, no relapse into sedation was reported after reversal with atipamezole. However, in the groups treated with an excessive dose of atipamezole compared to the optimal dose, heart rate increased beyond the baseline and decreased subsequently to the baseline level [32]. Yamashita et al. reported that the heart rate returned to the baseline level and then decreased after atipamezole administration in horses sedated with medetomidine and mentioned that similar changes were observed in horses treated with xylazine or clonidine [33]. The mechanism responsible for these tendencies remains unknown, although the administrations of atipamezole were predicted as the cause. It was a limitation of the present study.

When medetomidine is administered as a sedative, administration of atipamezole after sedation can reverse the tear-flow decrease caused by medetomidine. In practice, to prevent damage to the ocular surface, including the cornea, reversal with atipamezole as early as possible to recover tear flow is preferable, while attention should be paid to the fact that the analgesic effect of medetomidine is also reversed by atipamezole [34]. Protecting the ocular surface using ophthalmic gel or artificial tear solution is recommended rather than an overly early reversal with atipamezole when medetomidine is needed to exert an analgesic effect.

## 5. Conclusions

In conclusion, the optimal dose of atipamezole fully reversed the medetomidine-induced decrease in tear flow to the baseline level in rats. Though not fully, the lower dose of atipamezole recovered tear flow, while the higher dose of atipamezole reversed tear flow, but never beyond the baseline level. Our results suggest that a decrease in tear flow induced by medetomidine is a specific action via α_2_-adrenoceptor. However, it was not revealed which α_2_-adrenoceptor located in the central nervous system or peripheral tissues played a dominant role in the decrease in tear flow. To reveal the detailed mechanisms of this phenomenon, further investigations are required.
